# A Comprehensive Review on Temporal-Action Proposal Generation

**DOI:** 10.3390/jimaging8080207

**Published:** 2022-07-23

**Authors:** Sorn Sooksatra, Sitapa Watcharapinchai

**Affiliations:** National Electronic and Computer Technology Center, National Science and Technology Development Agency, Pathum Thani 12120, Thailand; sorn.soo@nectec.or.th

**Keywords:** temporal-action proposal generation, time-series analysis, proposal evaluation network, video descriptor

## Abstract

Temporal-action proposal generation (TAPG) is a well-known pre-processing of temporal-action localization and mainly affects localization performance on untrimmed videos. In recent years, there has been growing interest in proposal generation. Researchers have recently focused on anchor- and boundary-based methods for generating action proposals. The main purpose of this paper is to provide a comprehensive review of temporal-action proposal generation with network architectures and empirical results. The pre-processing step for input data is also discussed for network construction. The content of this paper was obtained from the research literature related to temporal-action proposal generation from 2012 to 2022 for performance evaluation and comparison. From several well-known databases, we used specific keywords to select 71 related studies according to their contributions and evaluation criteria. The contributions and methodologies are summarized and analyzed in a tabular form for each category. The result from state-of-the-art research was further analyzed to show its limitations and challenges for action proposal generation. TAPG performance in average recall ranges from 60% up to 78% in two TAPG benchmarks. In addition, several future potential research directions in this field are suggested based on the current limitations of the related studies.

## 1. Introduction

### 1.1. Background

Recent developments in computer vision have led to video analysis and understandings focusing on video categorization or captioning in academic and industrial fields. Since video content is generally based on motion information of objects and humans, their actions are the main key feature in video analysis and understanding. However, real-world videos (e.g., YouTube, Instagram, TikTok, and so on) are usually untrimmed. Therefore, temporal-action localization is one of the most important research topics in video understanding. Temporal-action localization focuses on action instances or proposals presented in untrimmed videos and categorizes their action classes and video content. Similar to object localization in an image, there are two fundamental processes in temporal-action localization consisting of temporal-action proposal generation (TAPG) and action recognition for localization and classification. As far as we are concerned, action recognition has shown significant progress and promising classification performance. In contrast, TAPG still has much room for improvement, especially in action proposal quality. Hence, several recent studies have aimed to improve TAPG to generate high-quality proposals to be utilized as input data for action recognition.

In general, TAPG techniques are utilized to classify whether the temporal proposals from untrimmed videos are background or action, and negative or positive results, respectively. There are two main techniques: anchor- and boundary-based techniques. The first technique generates candidate proposals with a pre-defined or fixed duration in multiple temporal scales. Then, a confidence score can be evaluated from each candidate. However, they are inflexible to various action proposal durations and have imprecise boundaries due to the fixed length of candidate proposals. Recently, a boundary-based technique was introduced to solve this problem in anchor-based methods. This technique was concerned with boundary prediction by utilizing the frame-level context information around the boundaries. Therefore, they could generate action proposals with a flexible duration and high accuracy of boundary prediction. Meanwhile, it was sensitive to noise and rich confidence scores from each candidate could not be extracted compared to anchor-based techniques. In addition, other researchers focused on how to optimize or train existing TAPG models to increase the accuracy of action boundary prediction. Semi- and weakly supervised learning [1,2] were discussed to minimize the size of training data and optimization time. Configuration of external modules (e.g., human detection, attention network, and so on) in the TAPG model [3,4] was also an important factor to improve prediction accuracy.

Although the TAPG model is similar to object localization (especially in region-based convolutional neural networks (R-CNN) [5]) for generating or localizing the candidate proposal, temporal localization has several challenges and differences, as follows:

1. High computation: as well as a spatial feature, TAPG also includes a temporal feature (1D temporal-series information), resulting in high computational cost.
2. Unclear boundary: Since there is no standard definition to distinguish between background and action proposals [6,7], proposal classification is based on personal opinions. It may be subjective to annotators and is ambiguous to both annotators and TAPG modeler/model designers. For example, drinking water could be started by pouring water into a cup or when water is put into the mouth.
3. Action class variation: There is the large number of action classes for handling several categories in action recognition. Since each action class has a specific characteristic (e.g., viewpoints, lighting condition, duration, and so on), it is unreliable to design a network for handling every action category.
4. Temporal length variation: Action proposals in untrimmed videos also have large variation in the temporal length. For example, shaking hands can only take a few seconds while a cooking process can take about 5 min. This causes the long-tail problem from imbalanced data. In addition, there are spatial variations similar to object localization (e.g., multi-sized objects, occlusion, and so on).

### 1.2. Survey Methodology

The related studies were initially searched using the keyword; ‘Temporal action proposal’, from 2012 to 2022. The three well-known databases—Scopus, Web of Science, and IEEE Xplore—are within the scope of this screening procedure. Most of the articles were selected from top-tier conferences in computer vision (e.g., AAAI, CVPR, ICCV, and so on). The scope of this review article is primarily determined by ‘temporal action proposal generation’.

**Figure 1:**
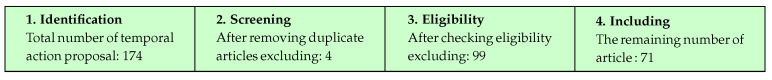
Block diagram of the article selection process.

**Figure 2:**
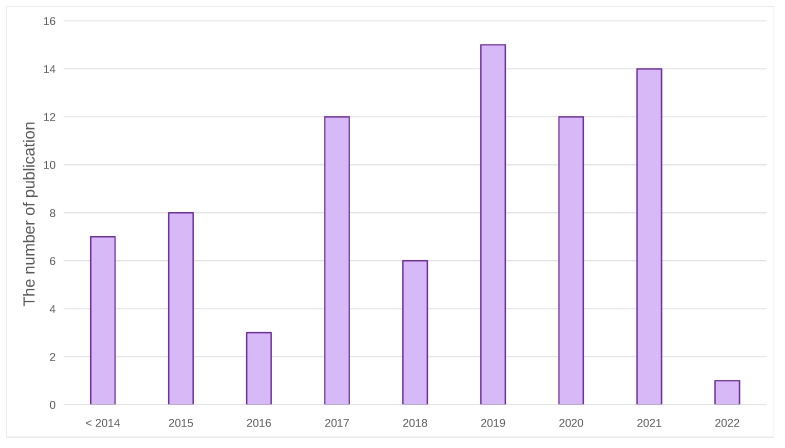
The number of publications from selected articles to the year of publication.

The article selection process is shown in Figure 1. There were four main steps, consisting of identification, screening, eligibility, and including. In the first step, the total number of related studies was 174. After finding duplicate articles, 4 articles are excluded. By considering only recent TAPG and its related work, only 71 were chosen for this evaluation. The selected publications are composed of three categories: conference proceedings (60.26%), journals (24.36%), and grey literature (arxiv) (15.38%). Figure 2 shows the number of these publications each year with the majority being in the past few years. After excluding irrelevant articles, 23 recent studies on TAPG were chosen by analyzing their significance in this area of research and evaluation criteria.

### 1.3. Contribution

This paper aimed to give a survey summarizing deep-learning-based techniques from recent studies on TAPG. Unlike a survey on temporal action localization [8], the related works in this review article focused on the proposal quality (e.g., confidence score, the proposal boundary accuracy, and so on) excluding action classification in their contributions. In addition, the proposal quality assessment was provided to obtain the advantages and limitations of related approaches.

### 1.4. Organization of the Work

**Figure 3:**
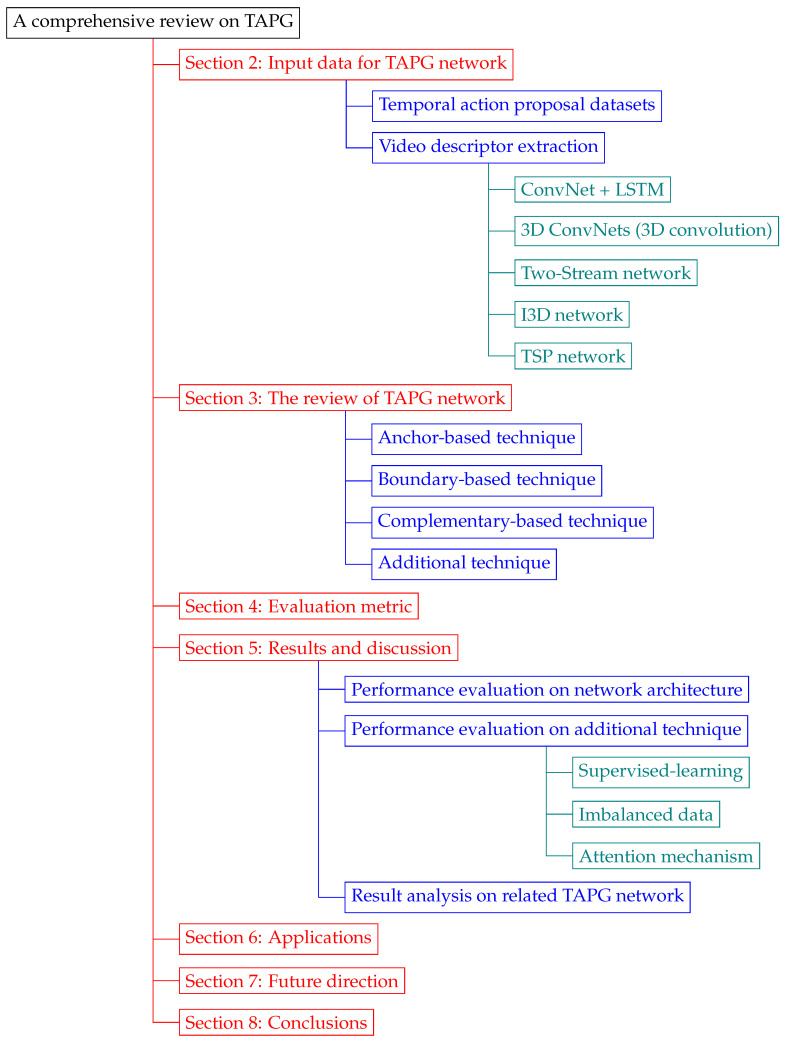
The organization of comprehensive review of TAPG.

As shown in Figure 3, the rest of this paper is organized as follows: Section 2 lists the input data for TAPG network, including temporal-action proposal benchmarks and video descriptors. Section 3 outlines the taxonomy for the TAPG model and its contribution. Section 4 describes how to evaluate the performance of proposal prediction models in various aspects. Experimental results from recent studies are analyzed and discussed in Section 5. The list of recent applications utilized by TAPG is provided in Section 6. Section 7 discusses the current challenges and suggests future directions. Section 8 concludes the review.

## 2. Input Data for TAPG Network

This section reviews the input data for video analysis from recent research related to TAPG. The input data could be categorized into two components: video data and descriptor. The video data can be downloaded from public datasets along with their action classes and temporal annotations. On the other hand, a video descriptor contains temporal and spatial features from an input video for feeding into the TAPG network. Therefore, the video dataset and the video-descriptor extraction can be summarized as follows:

### 2.1. Temporal-Action Proposal Datasets

As stated in the Introduction, the dataset for evaluating TAPG should have a high variation in video conditions (e.g., camera viewpoint, the number of action classes, temporal length, and so on) and provide untrimmed video annotation for temporal proposal localization. According to the Introduction, TAPG is one of the important components in temporal-action localization. The dataset related to temporal-action localization could be considered and evaluated in TAPG. The datasets’ videos were annotated with specific purposes for action localization and classification. The datasets were listed as follows:

THUMOS 2014 [9] was introduced to evaluate action recognition. This challenge was based on the UCF101 dataset [10] with video-level annotation consisting of 101 action classes in total. In 2014, there were 413 videos with temporal annotations for temporal-action localization. There were 200 and 213 videos for validation and testing, respectively. However, only 20 classes were provided in untrimmed videos. In addition, the ambiguous class was provided as the 21st action class in this dataset, where it represented unnatural motion or undecided action.

ActivityNet v1.3 [11] is a large-scale video benchmark for human-activity understanding. It is also used annually for a temporal-action detection challenge in CVPR. The dataset provided URL link for each videos. However, some of them could not be downloaded directly due to privacy restrictions or video removal by owners. All videos in ActivityNet v. 1.3 could be obtained by contracting the ActivityNet dataset official website. The total number of videos was 19,994 untrimmed videos consisting of 10,024, 4926, and 5044 videos for training, validation, and testing, respectively. The video duration was within 115 s on average. The number of classes was 200 categorized into 5 main classes with several subclasses. The average number of action proposals in each video was 1.54, where around 53.7 percent had a very short duration (0 to 30 s).

MEXaction2 [12] is a temporal-action proposal dataset focusing on two types of action, horse riding and bull–charge–cape. The number of video is 117 videos totaling 77 h from films produced between 1945 and 2011. Their videos were extracted from Institute National de l’Audiovisuel (INA), UCF101, and YouTube videos. The proposals from this dataset were categorized as 1336, 310, and 329 proposals for training, validation, and testing, respectively. Even though MEXaction2 has a moderate number of annotated videos, there is a limitation in terms of imbalanced data and high variability in point of view. In addition, videos extracted from old films have low resolution and are hard for classification.

MultiTHUMOS [13] presents a temporal-action proposal dataset with dense and multi-class annotated videos. It was updated from the THUMOS dataset. The total number of videos is 400 videos of 30 h with 38,690 temporal proposals and 65 action classes. Since it is the dense dataset, there are 1.5 labels per frame on average, and 10.5 action classes of each video. The MultiTHUMOS dataset is a challenging dataset for temporal-action proposal because there are many fine-grained action categories with low visual inter-class variation.

Charades [14] focuses on realistic activities without video editing or trimming. Their videos consist of 267 people recording videos in their own homes, of indoor activities. There are 9848 annotated videos with an average temporal length of 30 s and over 15% of them are multi-person interactions or activities. The videos from this dataset were categorized as 7985 and 1863 proposals for training, and testing, respectively.

AVA [15] is one of the large datasets providing both spatial and temporal positions for action proposal. It means that more than action annotations could be occurring in the same scene. There are 430 videos with 15 minutes average length and 80 action classes. With the various action labelings, there are 1.58 million action annotations in both spatial and temporal domains.

**Figure 4:**
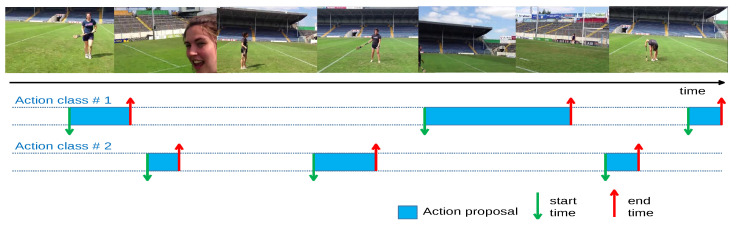
The example of untrimming dataset temporal annotation from ActivityNet v1.3 [11].

As far as we concerned, there are only two datasets (THUMOS 2014 and ActivityNet v1.3) utilized for TAPG evaluation in related studies due to a reasonable number of data annotations and high variation in action classes. In general, their temporal annotations were assigned as temporal boundaries (starting and ending time) for each action proposal, as shown in Figure 4.

### 2.2. Video Descriptor Extraction

Since input data from temporal-action proposal datasets are videos or frame sequences having their characteristics or video descriptors, their features should not contain only spatial features but also temporal features. Similar to image classification and object localization, deep-learning-based techniques were recently applied to this research. In recent research, the networks were designed for action recognition in untrimmed videos. Most TAPG researchers tended to adapt their architectures by utilizing the output scores of the top FC-layer. This network would later be trained by an action recognition network. Several models for extracting video descriptors were inspired by convolutional neural networks (CNNs) according to the following designs:

#### 2.2.1. ConvNet + LSTM

The image-classification network had a high performance for classifying spatial information. CNN could be modified for video classification that also had temporal information. The video features were extracted independently from each frame across the whole video [16]. To deal with temporal information, a recurrent layer (e.g., long short term memory (LSTM) [17,18]) was included. This layer was designed for encoding features from each frame as a sequence of spatial information. Therefore, CNN with LSTM helped to encode, state and capture both temporal ordering and long-range dependencies within the frame sequences. For network optimization, many researchers utilized cross-entropy loss on the output at every temporal position. In the testing process, the video descriptors could be extracted from the last layer of the CNN with LSTM from the last frame of the given video.

#### 2.2.2. 3D ConvNets (3D Convolution)

Since temporal information was considered as the third dimension of input data, 3D convolution [19,20,21,22] was a conventional approach to extract video descriptors similar to standard 2D convolutional networks. This network helped to directly visualize hierarchical representations of spatio-temporal data by using 3D kernels. However, there were issues with time complexity due to a larger number of model parameters from 3D convolutions. Although these methods aimed to deal with longer video durations, they processed only short sequences of fixed lengths ranging from 64 to 120 frames due to the limitations of computational cost and GPU memory. It was still non-trivial for these methods to learn from the entire video, due to their limited temporal coverage.

#### 2.2.3. Two-Stream Network

By utilizing LSTM or 3D convolution, actions with low-level motion might not be captured, while this motion was critical information in several cases. In addition, their networks required much time complexity from large models and ran through frame sequences. To solve these problems, the two-stream network [23] was designed by utilizing image and optical-flow classification networks represented as RGB and flow streams, respectively. It captured short temporal snapshots of videos by averaging predictions from a single image and a stack of 10 optical-flow images as input data or video snippets. Since an optical-flow image has two channels for horizontal and vertical directions, the flow stream has an adapted input convolutional layer with twice the RGB stream. Video features from each video snippet were extracted independently from the previous two-stream network [23], which supported only the short-range temporal structure. Since the video descriptor from the whole video could not be extracted, this method might not suitable for videos with various durations. Temporal segment network (TSN) [24] was designed for capturing features from the whole frame sequences with their modified two-stream networks. Unlike a traditional method, the segment consensus function was added as a post-processing step. All outputs in RGB and flow streams from each video snippet were fed into a segment consensus function to predict video descriptors with the relation among video snippets.

#### 2.2.4. I3D Network

The Inflated 3D ConvNets (I3D) network [25] was introduced by combining merits between two-stream networks and 3D convolutions with a series connection. In the two-stream network, the output data were generated frame by frame according to the RGB stream. On the other hand, the I3D network utilized 3D convolution to extract video descriptors over the frame sequence from the output features of two-stream networks. This combination helped to extract seamless spatio-temporal features for a video descriptor. Even though experimental results from the I3D network revealed a more promising performance than the two-stream network and 3D convolution, the pre-trained parameters for the two-stream network from the image domain were not optimized for 3D convolution in video tasks. Therefore, parallel inflated 3D convolution [26] was introduced to solve this matter. The inflated operation was regarded as an adaptation task rather than a pre-training process. In these networks, 3D convolution was treated as a 3D space container arranging multiple 2D convolutions in parallel to accommodate the neural connections of the 2D space.

#### 2.2.5. TSP Network

The structure of 3D convolution and the two-stream network were designed for extracting the spatio-temporal features of action recognition from datasets focusing on trimmed videos. In this case, it used only visual features from action temporal proposals, and background proposals were not considered in this case. Therefore, temporally sensitive pretraining (TSP) [27] was proposed for untrimmed videos, where features from background proposals were taken into account to improve temporal sensitivity. Since TSP was concerned with background and action proposals, two classification heads were utilized to train the encoder. The first head was similar to networks in trimmed videos where it helped to categorize action, including background proposals (without action). On the other hand, the main role of the second head was classifying the input frame sequence to be an action or a background proposal. Combining the results from the two heads, TSP is suitable for action localization in TAPG.

## 3. The Review of TAPG Network

Temporal-action proposal generation could be categorized into several categories depending on their purpose or scope of the study. In terms of network design, the TAPG network is categorized into anchor-based and boundary-based techniques, where their differences are temporal boundary precision and the confidence score on each proposal. In this section, the deep-learning-based methods for TPAG are reviewed by focusing on these two techniques. To fulfill the advantages and improve prediction performance, complementary techniques and additional techniques are also discussed. Hence, the related studies are categorized into four aspects, as follows.

### 3.1. Anchor-Based Technique

**Figure 5:**
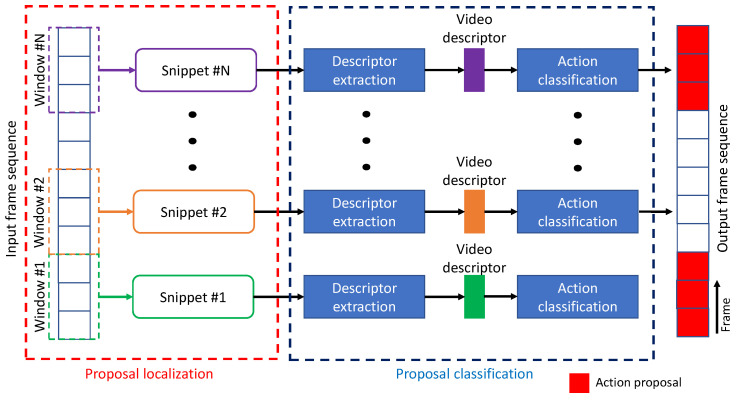
The general pipeline of an anchor-based technique.

This technique refers to the temporal refinements of pre-defined anchors with a fixed size of temporal length. In recent studies, the top-down approach was focused on enriching proposal features to generate their confidence scores. The whole scheme was similar to action recognition from trimmed videos, except the pre-processing was included to localize the proposal as the input for recognition. Therefore, there were two main stages, as shown in Figure 5, consisting of proposal localization and proposal classification. The first stage was to sample videos into several snippets, where non-overlapped temporal proposals were generated with the same temporal length. The last stage was utilized for extracting the video descriptor and classifying the class of each snippet, whether background or action proposals. The contribution summary for anchor-based techniques is shown in Table 1.

**TURN, 2017**: In the first technique, a sliding-window-based technique was deployed for proposal localization. Temporal Unit Regression Network (TURN) [28] was the traditional study for an anchor-based technique. Furthermore, 3D ConvNets (C3D [34]) was utilized as a video descriptor extraction. A video descriptor was extracted from each video snippet (every 16 frames) from the input video sequences. Clip pyramid was introduced to handle the variation in action proposals with various temporal lengths. There were two outputs generated by TURN, consisting of confidence scores and temporal coordinates determining whether the video snippet contained action and estimating the action boundaries (starting and ending time).

**Figure 6:**
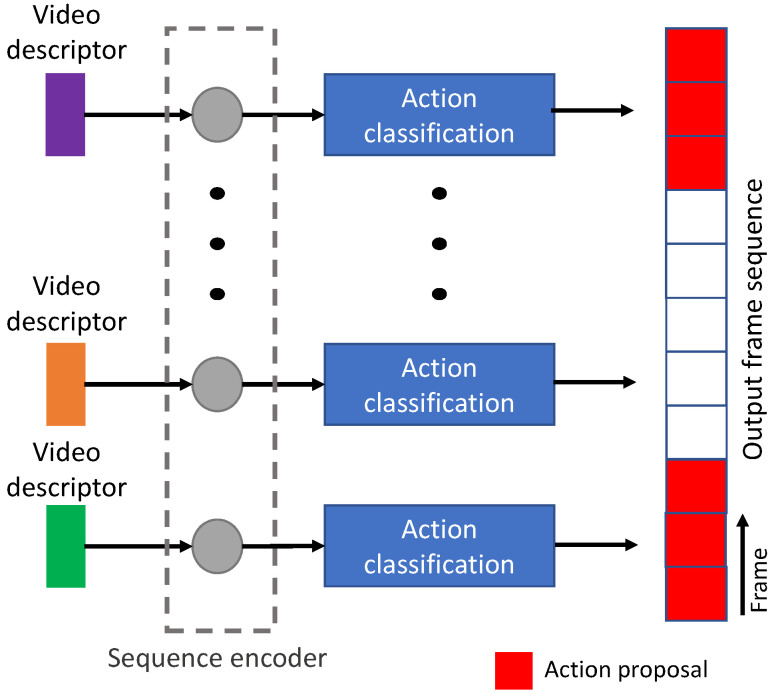
SST network architecture utilizing sequence encoder [29].

**SST, 2017**: Since a sliding-window technique might have dis-connectivity within the proposal or video snippet features and required high time complexity, Single-Stream Temporal (SST) action proposal [29] was introduced for extracting a video descriptor as a single stream without using sliding windows. To predict action proposals continuously, a recurrent model architecture was included to design a sequence encoder, as shown in Figure 6, for receiving C3D features [34] from the frame sequence (every 16 frames) of video data. In addition, their training process relied on a recurrent network with very long input video sequences without compromising model performance.

**SAP, 2018**: As a top-down approach, generating and searching action proposals through whole-video data generally requires a large time complexity and huge computational cost. Instead of focusing on every temporal position or video snippet in video data, the self-adaptive proposal (SAP) model [30] technique used a random search for action proposals by utilizing a sequence of transformations of the temporal window in random positions and covering action proposals that were as large as possible. The temporal positions were randomized by reinforcement learning based on the Deep Q-Learning algorithm [35]. Each video scenario was optimized independently. Moreover, a regression network was included to revise the offsets between predicted temporal proposals and the ground truth.

**SRG, 2019**: Untrimmed video characteristics usually contain several action proposals with similar action classes from each video snippet. It is well-known that temporal proposals with high recall and accurate boundaries could be generated as the overall performance. However, the proposal with specific action classes provided an unsatisfactory performance. This problem could be caused by the ignorance of relations among action proposals within untrimmed videos. To overcome this problem, Snippet Relatedness-based Generator (SRG) [31] presented snippet relatedness, to consider the local relations among temporal proposals. Snippet relatedness indicated that temporal positions were related to a specific action instance (e.g., jumping, walking, and so on). Therefore, the main score sequences included relatedness and starting and ending probabilities, where relatedness scores aimed to improve score sequence in specific action instances. To effectively learn the snippet relatedness, a pyramid non-local operation was also introduced to capture long-range dependencies among temporal proposals locally and globally.

**CMSN, 2019**: Action proposals from video snippets are usually continuous events and do not suddenly start or end. Therefore, the temporal stages close to the starting and ending time should be defined. The Continuous Multi-Stage Network (CMSN) [32] introduced a complete action-proposal stage consisting of ready, start, conform, end, and follow, occurring sequentially. There were six temporal stages, including background proposals. Ready and follow are the earlier and later stage of action proposals, respectively. Confirm is a stage between the starting and ending time. CSMN utilized ConvNet + LSTM [36] to encode video descriptors from video snippets. The feature was utilized to evaluate six stages of temporal proposals.

**RapNet, 2020**: Even though boundary-based techniques were effective in locating temporal boundaries, long-range contextual information was not taken into account, and an action with various temporal lengths was omitted. Relation-Aware Pyramid Network (RapNet) [33] presented a u-shaped architecture to generate temporal proposals from video descriptors via C3D [34]. To extract long-range contextual information, RapNet was enhanced by Relation-Aware Module, capturing bidirectional relations among local information as distilling context, to find a relationship between past and future content for augmenting multi-granularity temporal proposal generation. In addition, video-snippet confidence scores were considered to adjust the temporal boundaries as a post-processing step.

**Figure 7:**
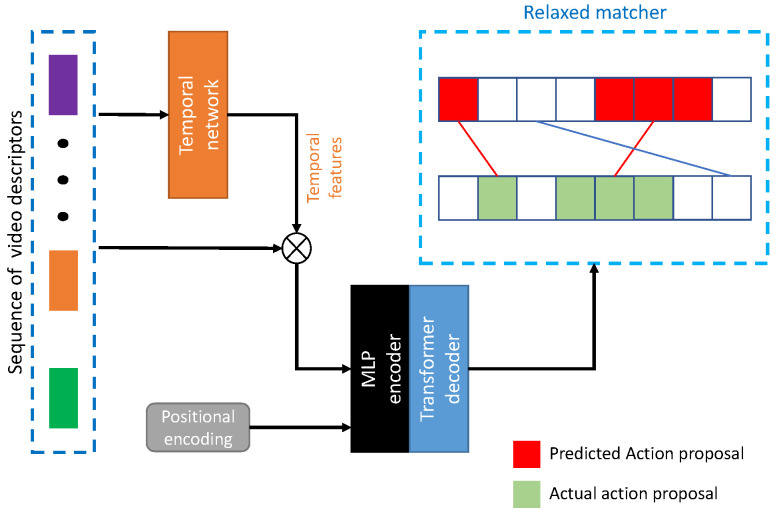
RTD-Net [3] architecture with relaxed matcher.

**RTD-Net, 2021**: Transformers were recently utilized in object detection with simpler architectures, smaller networks (in terms of the number of parameters), more throughputs, and quicker training. Since TAPG is similar to object detection, the transformer was able to predict temporal boundaries or proposals. Relaxed Transformer Decoders Network (RTD-Net) [3] firstly introduced TAPG with a transformer decoder modified by a vision transformer for object detection [37], as shown in Figure 7. The input of the transformer utilized temporal features extracted by a temporal network from each descriptor. This model was able to observe inter-proposal dependencies from global features and reduce computational cost. To overcome the over-smoothing effect from an encoder, the original transformer modified the boundary-attentive architecture with three prediction heads consisting of proposals, completeness scores, and classification scores. In addition, a relaxed matching scheme was introduced to relieve the strict criteria of a single assignment to each ground truth.

### 3.2. Boundary-Based Techniques

**Figure 8:**
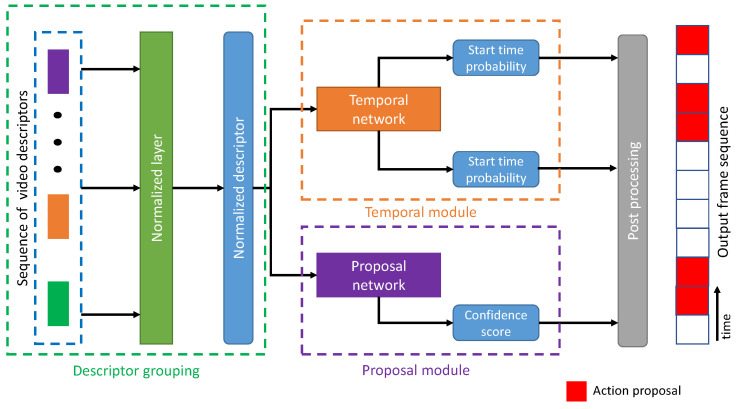
The general pipeline of a boundary-based technique.

As mentioned in the introduction, an anchor-based technique has a limitation on action proposals with various temporal lengths due to the fixed sizes of sliding windows. Even though pyramid-based or u-shaped architectures was utilized, the boundary precision from some action proposals, especially very short temporal length (less than 30 s), provided insufficient performance. Instead of predicting temporal positions, temporal boundaries (starting and ending time) were the focus of this technique which is considered a bottom-up approach to generate video descriptors before finding temporal information. Its general pipeline is categorized into three stages, as shown in Figure 8. The video descriptors from each snippet are normalized with specific sizes in the descriptor grouping. Then, this descriptor is fed into a classification module consisting of temporal and proposal modules. The first module generates the boundary probability sequence, then applies a boundary matching mechanism to generate candidate proposals in the second module. Finally, the post-processing step generates the action proposals. This technique helps to predict the probability of temporal boundaries and cover the flexible temporal duration of action instances. The contribution summary for boundary-based techniques are shown in Table 2.

**TAG, 2017**: The learning-based bottom-up scheme for TAPG was introduced in Temporal Actionness Grouping (TAG) [36] for forming confidence scores from video snippets through video data. This method was inspired by a watershed algorithm [44] to generate temporal proposals. The scheme was able to handle various video durations or a number of video snippets. Temporal Segment Network (TSN) [45], or a two-stream network, was utilized for extracting video descriptors from each snippet. Then, their features were encoded by TAG to generate confidence scores and distinguish between action and background proposals. All scores were grouped into temporal proposals of various granularities. Besides action and background proposals, the incomplete proposals were added as the third result for handling overlapping proposals among video snippets.

**BSN, 2018**: This research work was proposed to handle action proposal characteristics (e.g., flexible temporal length, precise temporal boundaries, and reliable confidence scores). Even though the first bottom-up approach was introduced by TAG [46], the temporal boundary was omitted in their TAPG model. Boundary Sensitive Network (BSN) [40] was designed to extract local and global features from normalized video descriptors for localizing temporal boundaries and retrieving candidate temporal proposals, respectively. Temporal boundaries (starting and ending time) from each candidate time were evaluated to generate starting, ending, and action probability sequences as local information. On the other hand, global information was obtained from proposal-level features via directly combining temporal locations with high starting and ending probabilities separately in a temporal module. Then, both pieces of information were combined as confidence scores in each candidate’s temporal proposals using a confidence module.

**BMN, 2019**: The current bottom-up proposal-generation methods utilize multiple stages to predict temporal boundary and confidence scores. However, they cannot efficiently generate adequately reliable confidence scores for retrieving proposals. Boundary-Matching Network (BMN) [41] was introduced as the first unified framework in bottom-up proposal generation methods for the confidence evaluation of densely distributed proposals. The temporal proposal could be denoted as a matching pair between starting and ending time from temporal proposals, which were combined as a 2D confidence map representing densely distributed proposals. The temporal and confidence modules were optimized jointly as a unified framework.

**DPP, 2019**: Even though the previous bottom-up approach succeeded in handling action proposals with various temporal lengths, their temporal proposal quality relied on action grouping or normalized video descriptors. In addition, their strategy was complex and time consuming. To solve this issue, Deep Point-wise Prediction (DPP) [38] was introduced as a simple yet efficient method that does not utilize any predefined sliding windows to generate temporal proposals. Inspired by the feature pyramid network [47], the model was designed for extracting temporal features in different temporal lengths or scales from low to high levels via a top-down pathway. Similar to other approaches, there were three main scores consisting of the confidence scores (action and background proposals), starting scores, and ending scores of different temporal lengths.

**DBG, 2020**: The previous boundary-based techniques predicted temporal boundaries as 1D information, starting and ending probabilities, in each temporal position. This strategy lacked global features for action proposals with blurred boundaries and various temporal lengths. Dense Boundary Generator (DBG) [39] was introduced to employ a global proposal feature and predict boundary maps as 2D information, where starting and ending probabilities were matched with both starting and ending time. Moreover, the base module was designed specifically for RGB and optical flow from video descriptor. The two-stream network [24] was utilized to extract the rich local temporal video representation as input, to exploit the rich local behaviors within the video sequence.

**BC-GNN, 2020**: Boundary-based techniques were utilized for finding starting and ending times. Thus, the relationship between action contents and their temporal boundaries could be defined. Therefore, Boundary Content Graph Neural Network (BC-GNN) [42] was proposed to implement predicted temporal proposals via a graph neural network, where temporal boundaries (starting and ending time) and action content were represented as a node and edge between them, respectively. Then, BC-GNN presented a novel graph computation operation for updating nodes and edges to predict the candidate temporal proposals and their confidence scores with higher quality.

**IntraC and InterC, 2020**: This research work [43] introduced a mechanism for the mutual regularization of temporal boundaries consisting of starting, continuing, and ending stages. These three stages were predicted as ordering triplets independently. However, other studies ignored their relations, leading to reduced temporal boundary precision, especially on input videos lacking sufficient discriminative information. Therefore, this framework focused on finding the relations among three stages in both the intra-phase and inter-phase. The intra-phase loss had the main role of reducing discrepancy inside each stage independently. On the other hand, the inter-phase loss was utilized for maintaining consistency among the three stages. Finally, these potential constraints could be realized by joint optimization between the intra-phase and inter-phase.

**ATAG, 2021**: Irrelevant frames from complicated action proposals were also an important issue in TAPG, where their frame-level proposal features had various characteristics and were hard to classify. However, only local proposal features were insufficient to deliver promising performance. To overcome this problem, Augmented Transformer with Adaptive Graph (ATAG) [4] utilized an augmented transformer with an adaptive graph network. A vanilla transformer [48] was utilized for generating action scores, especially on complicated action proposals. On the other hand, an adaptive graph network helped to exploit the temporal position and gradient information among temporal proposals or adjacent features, to classify local proposal features from irrelevant frames.

### 3.3. Complementary Techniques

With the merits of anchor- and boundary-based techniques, many researchers tended to propose complementary techniques between these two methods. A complementary technique is a combined module from the anchor- and boundary-based techniques to design the TAPG network. There was no standard network architecture for this technique. The contribution summary for complementary techniques are shown in Table 3. These networks were designed for a specific purpose, as described below.

**CTAP, 2018**: Even though a sliding window technique was able to cover all temporal segments in the videos, their predicted temporal proposals provided imprecise temporal boundaries. On the other hand, more precise temporal boundaries could be achieved by temporal and proposal modules in the boundary-based technique. However, temporal proposals with low confidence scores might be ignored. Therefore, Complementary Temporal Action Proposal (CTAP) [49] utilized complementary characteristics from the two techniques. A two-stream network [23] was utilized as video descriptors for both sliding windows and modules in a boundary-based method, where they were designed as a parallel structure. Since action proposals with low confidence scores could be omitted, this complementary structure helped to fill the omitted action proposals with a sliding-window method. Finally, the post-processing module helped to rank the predicted proposals and adjusted the temporal boundaries.

**MGG, 2019**: This technique [50] introduced the boundary-matching mechanism for densely distributed temporal proposals. Multi-Granularity Generator (MGG) was another TAPG utilizing complementary characteristics. The anchor- and boundary-based techniques designed a parallel architecture evaluating confidence scores and generating candidate temporal proposals, respectively. Instead of relying on a sliding-window technique, candidate temporal proposals were generated by utilizing a u-shaped architecture with lateral connections for handling various temporal lengths from each action instance. On the other hand, the middle time (besides starting and ending time) was taken into account for calculating action scores. Finally, the output from the two methods was combined as final temporal proposals.

**TRAN, 2021**: The TAPG with a transformer [52] was the focus of TAPG with transformer (TRAN) [51] to capture rich long-range dependencies. Anchor-based and boundary-based techniques were presented to capture frame- and proposal-level contexts, respectively. The overall network architecture was similar to other boundary-based techniques (e.g., BSN, BMN, and so on), but a temporal sliding window was utilized to generate candidate temporal proposals. Moreover, a sparse sampling mechanism was utilized to generate the sparse proposal sequence instead of the densely distributed proposals, which brought imbalanced data distribution between background and action temporal proposals.

### 3.4. Additional Techniques

As described above, many studies tended to focus on the modification of the TAPG network to improve classification performance. However, other studies aimed to propose techniques to solve issues related to data such as the amount of annotated data, imbalanced data, and the characteristics of the TAPG dataset. Since annotating video sequences requires a large amount of time and labor effort, semi-supervised learning was taken into account to reduce the amount of annotated data [2]. In addition, temporal length might vary greatly, resulting in a large variation in temporal proposals called a long-tail problem. The weighted loss function and re-sampling training data were used to reduce the effect of imbalanced data. In addition, a characteristic of several TAPG datasets was that the action was usually performed by a human. With respect to this, an attention model focusing on human regions could be utilized for extracting salient features. The following section discusses additional techniques related to the specific characteristics of the TAPG dataset:

**Supervised learning**: To achieve high temporal-boundary prediction accuracy, supervised-learning-based techniques were utilized by recent TAPG networks. However, these techniques required a large amount of annotated temporal action intervals from untrimmed videos. On the other hand, unsupervised learning-based techniques [53] (without data annotation for training) usually have a poor overall performance for generating temporal proposals. Therefore, the semi-supervised-based technique was firstly introduced in this study [1]. With a small amount of annotated training data, this technique helps to achieve label efficiency. Mean Teacher [54] was modified for a semi-supervised framework. The model consisted of time warping and time masking for providing perturbations for time-sensitive tasks and optimizing models to be more robust and generalize better to unseen data.

Even though the semi-supervised learning technique was a well-known method for minimizing the number of annotated temporal action intervals from untrimmed videos, the perturbations ignored the temporal interactions, which were critical for learning robust action representations. This issue could be solved by using a fully non-annotated temporal action proposal. Therefore, self-supervised learning was the focus of this research [2], to fulfill proposal features for non-annotated video data on TAPG with semi-supervised learning using the Mean Teacher model [54]. Their model, based on self-supervised learning, designed two simple perturbations for temporal-feature shifting [55] and temporal-feature flipping. The first module had a role to move some randomly selected channels of feature maps. The overall features were flipped by the second module.

**Imbalanced data**: Despite the improvement in temporal proposal generation, the failed experimental cases were not deeply analyzed, especially in action proposals with various temporal lengths. According to this research [56], several actions with short temporal lengths were missing in the previous works, where this problem was caused by imbalanced temporal lengths in the TAPG benchmark. To solve this problem, the scale-invariant loss was proposed for confidence regression to reduce the effect of a low sample of action with a short duration. In addition, the module for predicting temporal boundaries included a u-shaped architecture to enhance global proposal features with higher precision.

Besides action temporal length, there was an imbalance between the number of positive and negative samples. The two-stage re-sampling [57] was presented to balance positive–negative samples and samples with various temporal lengths. Their experiment utilized a modified version of BSN (BSN++) for evaluation. Moreover, the u-shaped architecture included nested skip connections to capture rich contexts in temporal boundaries. In addition, a bi-directional boundary matching mechanism was introduced, where reversing video descriptors were utilized as input data. In general, starting time could be found by observing a sudden change from background to action proposals. By using video descriptors with reversed directions, a sudden change in the opposite direction could be handled by the various types of action proposals.

**Figure 9:**
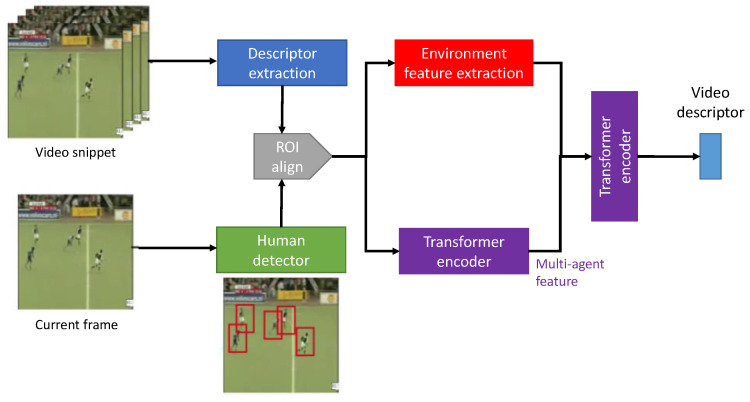
The architecture of AEN [58] with transformer encoder for extracting a video descriptor of each video snippet.

**Attention mechanism**: Since actions in public videos are usually performed by humans, the TAPG network should be focused on the spatial and temporal region containing the human body. To realize this concept, Agent-Environment Network (AEN) [58] developed a prepossessing step to localize humans or agents performing an action within action proposals by the human detector, as shown in Figure 9. Moreover, an environmental pathway was introduced to visualize how agents interacted with the environment, helping action classification.

## 4. Evaluation Metric

TAPG evaluation is considered as a binary classification between action and background proposals, which are positive and negative samples, respectively. There are four parameters for TAPG evaluation, consisting of true positives (TP), false positives (FP), true negatives (TN), and false negatives (FN). The relations for these parameters are shown in Table 4. The intersection of union (IoU) in the temporal domain is taken into account for evaluating the TAPG network because TAPG is similar to object detection in a spatial domain. In the temporal domain, IoU is considered as the overlap between predicted and actual temporal proposals (both action and background proposals) by the TAPG network and ground truth, respectively. IoU can be calculated by Equation (1).

(1) $$IoU=\frac{P_{p}\cap P_{a}}{P_{p}\cup P_{a}}$$

where $P_{p}$ and $P_{a}$ are predicted and actual proposals, respectively. IoU is used for checking temporal localization accuracy from predicted proposals. Without action classification, the number of the predicted proposal is the only focus. Therefore, related studies tended to utilize recall (*R*) and average recall (AR) for evaluation. A recall is the coverage of correct prediction. Specifically, a recall is how many actual positive samples in the testing set are identified. AR is an average recall over the number of predicted proposals ordered by their confidence scores (from high to low). The calculation formulas of *R* and AR are depicted in Equations (2) and (3)

(2) $$R=\frac{TP}{TP+FN}$$

(3) $$AR_{A_{N}}=\frac{\sum_{i}^{A_{N}}R_{i}}{A_{N}}$$

where $A_{N}$ is an average number of predicted proposals. In an evaluation procedure, the TAPG network is tested from 1 to 100 $A_{N}$. In general, AR@100 is used as the primary evaluation metric in most recent related works. To classify recall, IoU is utilized as a threshold which has range of [0.5: 0.05: 0.95] and [0.5: 0.05: 1.0] for ActivityNet and THUMOS, respectively. Moreover, the area under the curve (AUC) between AR and $A_{N}$ is calculated to check the total performance of the TAPG network.

## 5. Results and Discussion

According to the TAPG evaluation metric, most related studies were concerned about the quantity of predicted temporal proposals. Therefore, $AR@A_{N}$ was utilized in their evaluation. In THUMOS 2014, their videos were evaluated with various numbers of predicted proposals (from $A_{N}$ = 50 to $A_{N}$ = 1000), as shown in Table 5. However, the evaluation of ActivityNet focused on overall performance with AR@100 and AUC due to the large amount of video data, as shown in Table 6. The related studies have different scopes and purposes; therefore, the network evaluations have different aspects, as follows:

### 5.1. Performance Evaluation on Network Architecture

As mentioned in Section 3, the proposed TAPG network architecture was categorized into the anchor-, boundary-, and complementary-based techniques. Their empirical results are depicted in Table 5 and Table 6 on various video descriptors. Table 5 indicates that AR is increased with higher $A_{N}$ or the larger number of predicted proposals. The recent experimental results showed that the recent boundary-based technique had more AR than anchor-based techniques by focusing on the temporal boundary. However, the most effective TAPG network utilized the merit between these two techniques. It showed that the complementary technique should be considered and further developed, where the effective networks were aided by a transformer (e.g., RTD-Net, ATAG, and TRAN) or attention networks (e.g., RapNet). In terms of the video descriptor, the two-stream network was utilized and had more AR than other descriptors (C3D and I3D).

Without data annotation in test video data from ActivityNet, the related studies evaluated their networks with validation video data as shown in Table 6. Unlike the THUMOS dataset, the recent anchor-based techniques were more effective than boundary-based techniques due to the characteristics of video data in ActivityNet. Even though the boundary-based technique was designed for various temporal lengths on action proposals, the action proposal with a very short temporal length (less than 30 s) was ineffective for their boundary prediction. This problem is still a challenge for this dataset because 53.7% of action proposals have a very short temporal length. This limitation of the boundary-based technique also dropped the performance of a complementary technique. Even though a two-stream network was utilized for the descriptor extraction from most related studies in ActivityNet, the results from 3D convolution had higher AR with the same TAPG network architectures (e.g., RapNet and BSN).

### 5.2. Performance Evaluation on Additional Techniques

This section described the performance evaluation of the network optimization techniques in the TAPG network. The related studies used additional techniques for the TAPG network to solve following problems: supervised learning [1,2], imbalanced data [56,57], and attention mechanism [58]. To evaluate the performance in those problems, BMN [41] was selected to be utilized as the TAPG network for performance comparison as follows.

#### 5.2.1. Supervised Learning

**Figure 10:**
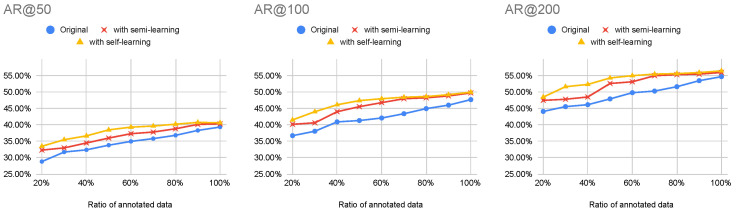
The AR@AN from BMN using the original two-stream network [41], with semi-learning [1], and with self-learning [2].

The experimental results evaluating semi-supervised learning [1] and self-supervised learning [2] are shown in Figure 10. This experiment utilized BMN as the TAPG network with the two-stream network. The results indicated that the performance with a lower amount of annotated data obtained a lower value in $AR@A_{N}$. On the other hand, semi-supervised learning helped to increase an average recall in every $A_{N}$ and ratio of annotated data. By focusing on the non-annotated data, self-supervised learning outperformed semi-supervised learning.

#### 5.2.2. Imbalanced Data

Since the imbalanced data of temporal action lengths was one of the main challenges (especially in ActivityNet), scale-invariant (SI) loss from [56] was evaluated using BMN and DBG, as shown in Table 7. In their experiment setup, the temporal length of action proposals was categorized by the ratio (*s*) of their video duration consisting of short (0.0 ≤ *s* < 0.06), medium (0.06 ≤ *s* < 0.6), and long (0.6 ≤ *s* < 1.0). Table 7 shows that the TAPG network without SI loss was ineffective to action with short temporal length. There was an improvement by using SI-loss in both BMN and DBG of around 1% and 4%, respectively. Specifically, because the imbalance issue in DBG was not as severe as in BMN, AUC gains for DBG are not as much as BMN. However, it was still acceptable. In average scales, it proved that networks with SI-loss outperformed their original versions.

#### 5.2.3. Attention Mechanism

This section evaluated effects of an attention mechanism focusing on the agent or human regions proposed by AEN [58]. The THUMOS 2014 was analyzed as shown in Table 8. Their empirical results indicated that overall performance was improved by extracting features from specific regions of interest. However, some action proposals without an agent might be ineffective in this case, although this issue was not mentioned in their experiment.

### 5.3. Result Analysis on Related TAPG Network

This section discusses the result of our evaluation. Even though there is a standard evaluation criterion mentioned in Section 4, other aspects should be discussed. As the most promising performance in Table 5, a boundary-based technique was selected for our analysis. In our experiment, BMN [41] was implemented and evaluated by using a public open-source code (https://github.com/JJBOY/BMN-Boundary-Matching-Network) (accessed on 2 August 2021) on ActivityNet v1.3 [11]. We found several limitations, summarized below, that should be improved upon in future research.

#### 5.3.1. The Temporal Proposal with Similar Pattern

**Figure 11:**
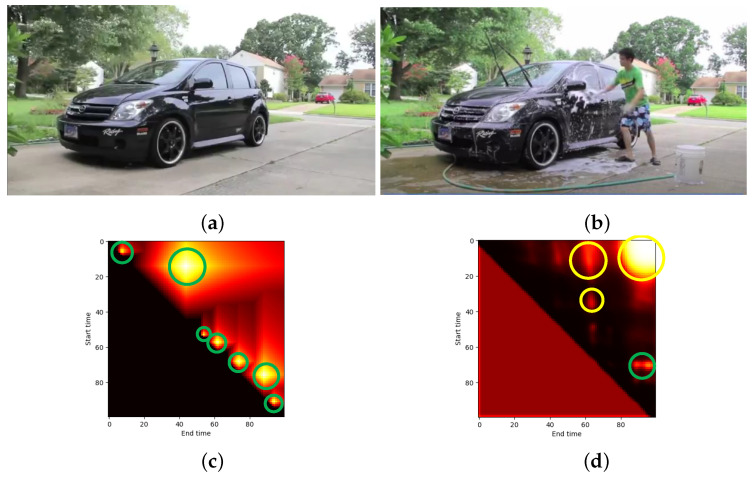
The example video with similar background from ActivityNet [11] consisting of (**a**) background proposal, (**b**) action proposal, (**c**) actual and (**d**) predicted confidence map, where green and yellow indicate the correct and wrong temporal locations of action proposal, respectively.

**Figure 12:**
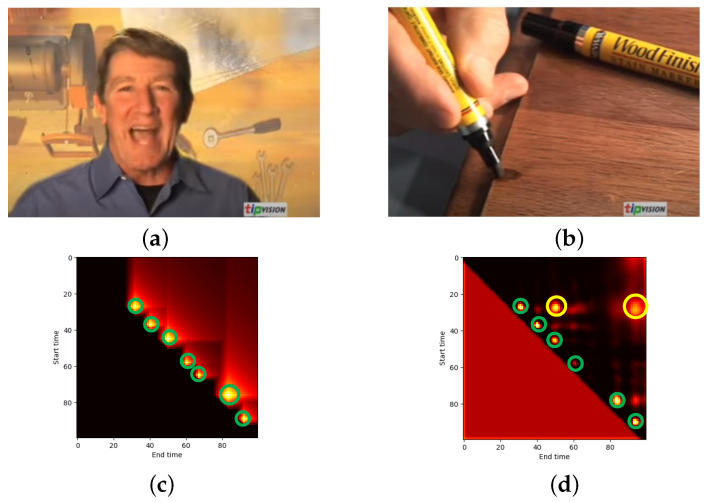
The example video without similar background from ActivityNet [11] consisting of (**a**) background proposal, (**b**) action proposal, (**c**) actual and (**d**) predicted confidence map, where green and yellow indicate the correct and wrong temporal locations of action proposal, respectively.

In general, proposals with similar patterns (e.g., viewpoint, background, and so on) are classified as the same classes (action or background proposal). If action and background proposals have similar patterns, it might cause false positives in predicted proposals and generate imprecise action boundaries as the result. To visualize this hypothesis, the confidence maps from examples with and without similar patterns between action and background proposals are shown in Figure 11 and Figure 12.

Figure 11 shows the confidence maps (bottom row) from a video with similar patterns (Figure 11a,b). The actual confidence map from Figure 11c indicated the action proposal within the green circles. However, the predicted confidence map (Figure 11d) was different to its ground truth. The TAPG model tended to focus on background proposals (yellow circles) and some action proposal scores (green circles) were suppressed. On the other hand, a video with a different pattern for the background (Figure 12a) and action proposals (Figure 12b) was effective for the TAPG model. The actual confidence map (Figure 12c) was similar to its predicted map (Figure 12d). It indicated that the TAPG model was able to localize the action proposals more precisely. Therefore, the pattern between action and background proposals is one of the main factors for classifying proposals and the TAPG model should be concerned about this problem.

#### 5.3.2. Contiguous Action-Proposal Merging

According to various temporal lengths, action proposals with long temporal lengths were effective in the TAPG model. However, the proposals with short temporal lengths were ignored. As mentioned in Section 5.2.2, this problem was caused by the imbalance of data in temporal lengths. On the other hand, it can be affected by the proposal merging from a group of contiguous proposals. If the temporal boundaries are too close to each other, the TAPG might merge two or more proposals as one proposal with a longer temporal length.

**Figure 13:**
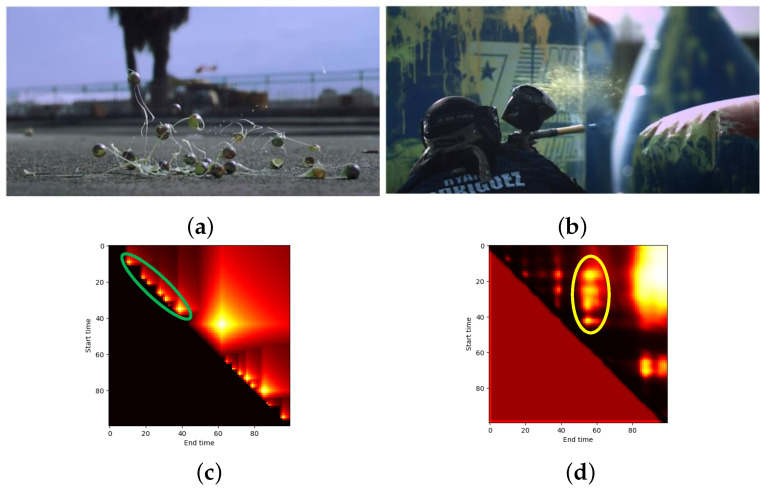
The example video contiguous action proposals from ActivityNet [11] consisting of (**a**) background proposal, (**b**) action proposal, (**c**) actual and (**d**) predicted confidence map, where green and yellow indicate the group of action proposal and their merging proposals, respectively.

Figure 13 shows the example of videos (Figure 13a,b) with contiguous action proposals and their confidence maps by our experiment. As you can see in Figure 13c, there was a group of contiguous action proposals within a green circle. However, their predicted scores in Figure 13d were focused between their earliest start time and the latest ending time. It indicated that there was a merged proposal by a TAPG model. Therefore, contiguous proposals and proposal merging should be considered, to solve actions with various temporal lengths, especially in action proposals with short temporal lengths.

## 6. Applications

In practical applications, TAPG had the main role of extracting action proposals from input video data. As described in the Introduction, these action proposals were mainly utilized as the pre-processing step for trimming videos in various applications (e.g., temporal action localization (TAL) [8,59,60,61], video retrieval [62,63,64], video highlight detection [65,66,67], video summarization [68,69], and so on). These applications and their related works were briefly described, as follows.

### 6.1. Temporal Action Localization

**Figure 14:**
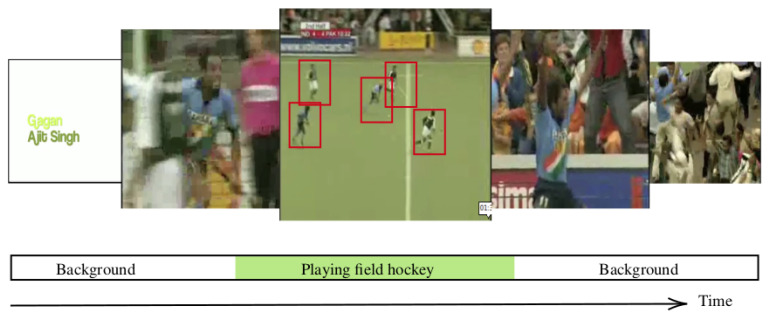
Example of temporal and spatial positions (within red rectangular boxes) by TAL from ActivityNet [11].

Different from TAPG, TAL was an extended work of TAPG including action recognition. TAL aimed to localize the temporal position of each action class (Figure 14), where TAPG helped to extract candidate proposals. The output action classes were dependent on their scopes of studies. Daily-life activity from ActiviyNet and THUMMOS was the focus of the research to handle public videos. The SlowFast network [59] was proposed to focus on the variations in action speed. Specific incident or accident detection was discussed by Rendon-Segador et al. [60]. In addition, the TAL could be developed for extracting the spatial locations [61] of each action class, similar to object detection, as shown in the red rectangular boxes in Figure 14.

### 6.2. Video Retrieval

Video retrieval is the approach for facilitating the video search of large public video collections by indexing their contents or categories. Without examining the video contents, video search relies on metadata such as captions or keywords. TAPG could be utilized in video retrieval for extracting relevant frames or proposals from their video captions and contents. The video retrieval is generally divided into two major modules (feature extraction and similarity model). The similarity model is the main part for checking the similarity of visual features from video contents in the database.

### 6.3. Video Highlight Detection

Besides action detection and classification, video highlights are another important process for video categorization or video search with similar techniques. TAPG can be modified for detecting highlight proposals, capturing a user’s primary attention within an untrimmed video, especially in a sports video. This application has the potential to significantly ease video search. The highlight is generally cued by observing or extracting latent signals from their benchmarks, depending on user’s demands or perspectives. Weakly supervised learning [65] is considered in order to minimized time complexity because this application has a large number of video categories.

### 6.4. Video Summarization

Video summarization is an application for generating a short synopsis and excluding unnecessary content. It helps to summarize untrimmed video data by selecting its informative or key frames. The output from a video summarization usually consists of a set of video frames (video-key-frames), ordered by time [68]. As mentioned in the Introduction, an action was considered as the main key feature for video analysis and understanding. Action proposals could contain the key frames for video summarization. Similar to video highlight detection, the type of video-key-frames are several and there is a large number of video categories. Currently, weakly supervised learning is the main focus of this research.

## 7. Future Direction

As discussed in previous sections, limitations can be overcome for future directions in TAPG. This section discusses several interesting directions from the related experimental results. The future trends may be as follows:

### 7.1. Non-Prior Knowledge Attention Mechanism

**Figure 15:**
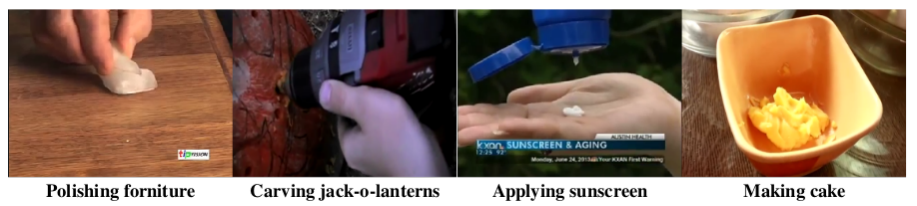
The examples of action proposals without human body part from ActivityNet [11].

Vision attention mechanisms have received much attractive throughout the last decade. As mentioned in AEN [58], the action should be performed around the agent or human regions. An attention mechanism was taken into account to extract their agent features. However, action proposals might exclude the human body, as shown in Figure 15. These action proposals focused on the specific object in the scene instead. Without agents, these proposals might be ignored by AEN. In addition, the problem on merging of contiguous action proposals might be an focus of this research. In addition to focusing on agents or objects, attention mechanisms focusing on the temporal domain should be discussed to emphasize contiguous action proposals, especially on short proposals. Therefore, the new attention mechanism should not rely on a prior-knowledge guide learning to handle all the circumstances of action proposals. In addition, a vision transformer for self-attention [70] should be applied to solve this issue in the future.

### 7.2. Joint Learning of Audio Information

**Figure 16:**
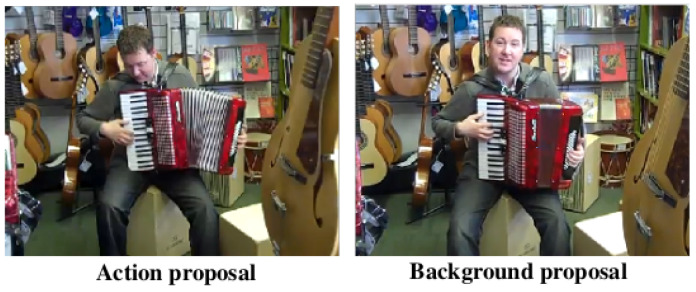
The examples of action (**left**) and background (**right**) proposals from playing an instrument in ActivityNet [11].

Audio information could be considered as a relevant feature in action recognition and classification. However, only image information was taken into account in recent studies on TAPG. By observing videos in the benchmarks, the characteristics between action and background proposals (Playing accordion) are ambiguous and hard to distinguish, as shown in Figure 16. Even though they have similar postures, the person in the scene does not play music in the background proposal. To handle this problem, audio information should be included. Recently, related studies have leveraged the natural synchronization between image and audio to learn an acoustic representation [71]. It means that the audio information from the action and background proposal could be distinguished from their acoustic representation. Therefore, audio information will be salient information for video analysis in the future direction.

### 7.3. New Video Descriptor

A video descriptor is one of the most important factors for predicting action proposals. As far as we are concerned, TSP [27] was the only proposed video descriptor designed for TAPG and TAL. Besides action proposals, TSP also extracted features from background proposals or negative samples to increase TAPG performance. Even though TSP has a higher performance in AR than other video descriptors from their experiment [27], this descriptor was rarely utilized in several related studies due to a fair improvement. The related studies tended to utilize public and extracted video descriptors. Therefore, several descriptors should be discussed in future research for obtaining their strengths and weaknesses. Then, more video descriptors can be proposed to overcome the previous video descriptors specifically designed for the TAPG network, as another future work.

## 8. Conclusions

This paper conducted a comprehensive review of deep-learning-based techniques for temporal-action proposal generation. Related studies were discussed including video descriptors and their TAPG networks, consisting of anchor-based, boundary-based, and complementary techniques. Besides the network architecture, additional techniques were also included. Recent TAPG studies, including its benchmark, were selected from specific keywords of TAPG and evaluation metrics during 2012 and 2022. The empirical results showed that the two-stream network yielded higher performances in average recall in both TAPG benchmarks (AcitvityNet v1.3 and THUMOS 2014). Based on our result analysis, there were two main weak points in recent work consisting of similar patterns among proposals and contiguous proposal merging. In addition, the various aspects of the predicted action proposals were discussed for a specific purpose. Over the past few years, the related studies on TAPG tended to focus on the attention mechanism to extract salient features via the attention module and self-attention from the vision transformer.

Since TAPG is expected to play a significant part in temporal action localization and video analysis, TAPG networks should generate a promising performance (at least 90% in AR@100) for practical use. The TAPG network should be proposed and designed for specific action categories. Since untrimmed videos is the main input data for TAPG, video descriptor and attention modules should be redesigned instead of being adapting from other research (e.g., trimmed video classification, object detection, and so on). In addition, all information from a video, including audio, could be utilized to distinguish temporal proposals, especially on action proposals with sound such as playing musical instruments. Therefore, TAPG still has a gap for improvement on several complicated and challenging issues as future directions.

## Figures and Tables

**Table 1:** Contribution summary of anchor-based techniques.

	Ref.	Contribution
Proposal localization	[28]	- Introducing regression network for extracting video features in long untrimmed videos (every 16 or 32 frames).
	[29]	- Proposing non-overlapping video-descriptor extraction though whole-video data without relying on the sliding-window technique or sampling the video into multiple small video snippets.
	[30]	- Randomly localizing the action proposals while ignoring unnecessary temporal proposals.
	[31]	- Introducing the snippet relation score to recognize temporal proposals within a specific action class.
	[32]	- Presenting more action-proposal stages to handle the variation in action class and temporal length.
Proposal classification	[30]	- Adapting reinforcement learning for the TAPG network.
	[31,33]	- Utilizing a pyramid network as a distilling mechanism to capture video features with various temporal lengths.
	[33]	- Analyzing the bidirectional relations among temporal proposals by relation-aware module.
	[3]	- Adapting the transformer for object detection to visualize the global view of an action proposal and reduce time complexity.
Post-processing	[32]	- Proposing variable margin cosine loss to increase temporal-boundary localization accurately.
	[3]	- Generating the proposals via a relaxation mechanism to reduce ambiguous characteristics between action and background proposals.

**Table 2:** Contribution summary of boundary-based techniques.

	Ref.	Contribution
Descriptor grouping	[36]	- Introducing action confidence grouping to discriminate between complete and incomplete proposals.
	[38]	- Estimating the specific points from video descriptors to predict temporal boundary and confidence score and minimize time consumed.
	[39]	- Redesigning from BMN and BSN as 2D information between starting and ending time.
Classification module	[40]	- Designing temporal and proposal modules to handle the ’local to global’ fashion for boundary and proposal prediction, respectively.
	[39,41]	- Jointly unifying framework between temporal and proposal modules.
	[39]	- Introducing an auxiliary supervision via confidence-score classification.
	[4,42]	- Including a graph neural network to display a relationship between temporal and proposal modules.
	[43]	- Mutually regularizing the learning process of intra- and inter-phase consistency of action temporal proposals
	[4]	- Augmenting the transformer to capture long-range dependencies in a classification module.
Post-processing	[41]	- Generating densely temporal proposals by temporal-boundary matching mechanism.

**Table 3:** Contribution summary of complementary techniques.

	Ref.	Contribution
Descriptor grouping	[49]	- Designing boundary adjustment and proposal ranking network from sliding windows to retrieve proposals ignored by boundary-based techniques.
Classification module	[50]	- Including the anchor-based technique in the classification module with parallel architecture.
	[51]	- Modifying temporal and proposal modules from BSN and BMN with a vision transformer.
Post-processing	[50]	- Integrating scores from the confidence module and anchor-based technique to enrich the proposal quality.
	[51]	- Including the sliding-window technique to generate sparse multi-scale temporal proposals.

**Table 4:** The logic relation of binary classification parameters.

Prediction/Actual Results	Action Proposal	Background Proposal
Action proposal	True positive (TP)	False positive (FP)
Background proposal	False negative (FN)	True negative (TN)

**Table 5:** TAPG network comparison on THUMOS 2014 using $AR@A_{N}$.

Model Name	Video Descriptor	AR@50	AR@100	AR@200	AR@500	AR@1000
Anchor-based techniques						
TURN, 2017	C3D	19.63%	27.96%	38.34%	53.52%	60.75%
	Two-stream	21.86%	31.89%	43.02%	57.63%	64.17%
SST, 2017	C3D	19.90%	28.36%	37.90%	51.58%	60.27%
SRG, 2019	Two-stream	41.19%	49.72%	56.71%	63.78%	x
CMSN, 2019	C3D	40.40%	46.71%	52.46%	x	x
	Two-stream	43.77%	50.55%	56.48%	x	x
RapNet, 2020	C3D	29.72%	37.53%	45.61%	55.26%	61.32%
	Two-stream	40.35%	48.23%	54.92%	61.41%	64.47%
RTD-Net, 2021	I3D	41.52%	49.32%	56.51%	62.91%	x
Boundary-based techniques						
TAG, 2017	Two-stream	18.55%	29.00%	39.61%	x	x
BSN, 2018	C3D	29.58%	37.38%	45.55%	54.64%	59.48%
	Two-stream	37.46%	46.06%	53.21%	60.64%	64.52%
	I3D	36.73%	44.14%	49.12%	52.26%	x
BMN, 2019	C3D	32.73%	40.68%	47.86%	56.42%	60.44%
	Two-stream	39.36%	47.72%	54.72%	62.07%	65.49%
	I3D	37.03%	44.12%	49.49%	54.27%	x
DPP, 2019	C3D	25.88%	34.79%	43.37%	x	x
DBG, 2020	C3D	30.55%	38.82%	46.59%	56.42%	62.17%
	Two-stream	37.32%	46.67%	54.50%	62.21%	66.41%
BC-GNN, 2020	C3D	33.31%	40.93%	48.15%	56.62%	60.41%
	Two-stream	40.50%	49.60%	56.33%	62.80%	66.57%
ATAG	C3D	34.47%	41.92%	49.60%	58.49%	62.24%
	Two-stream	43.52%	51.86%	59.48%	66.04%	70.28%
IntraC & InterC, 2020	I3D	44.23%	50.67%	55.74%	x	x
Complementary techniques						
CTAP, 2018	Two-stream	32.49%	42.61%	51.97%	x	x
MGG, 2019	C3D	29.11%	36.31%	44.32%	54.96%	60.98%
	Two-stream	39.93%	47.75%	54.65%	61.36%	64.06%
TRAN, 2021	Two-stream	43.90%	53.50%	60.20%	66.70%	68.80%

**Table 6:** TAPG network comparison on ActivityNet ver 1.3 using $AR@A_{N}$ and AUC (Val).

Model Name	Video Descriptor	AR@1	AR@100	AUC
Anchor-based techniques				
TURN, 2017	C3D	x	49.74%	54.16%
SRG, 2019	Two-stream	x	74.65%	66.06%
RapNet, 2020	C3D	x	78.63%	69.93%
	Two-stream	x	76.71%	67.63%
RTD-Net, 2021	Two-stream	33.05%	73.21%	65.78%
Boundary-based techniques				
BSN, 2018	C3D	x	75.61%	67.47%
	Two-stream	32.17%	74.16%	66.17%
BMN, 2019	Two-stream	x	75.03%	67.10%
	TSP	34.99%	76.63%	69.04%
DBG, 2020	Two-stream	x	76.65%	68.23%
BC-GNN, 2020	Two-stream	x	76.73%	68.05%
ATAG, 2021	Two-stream	x	76.75%	68.50%
IntraC & InterC, 2020	I3D	x	75.27%	66.51%
Complementary techniques				
CTAP, 2019	Two-stream	x	73.17%	65.72%
MGG, 2019	Two-stream	x	74.54%	66.43%
TRAN, 2021	Two-stream	34.90%	76.50%	68.30%

**Table 7:** The ablation study of scale-invariant (SI) loss [56] from BMN [41] and DBG [39] with the two-stream network using AUC in ActivityNet.

Methods	0.0 ≤ *s* < 0.06	0.06 ≤ *s* < 0.65	0.65 ≤ *s* < 1.0	Average Scales
BMN	36.53%	70.43%	94.48%	67.10%
BMN with SI-Loss	40.24%	70.32%	94.41%	67.98%
DBG	39.07%	72.18%	93.08%	67.90%
DBG with SI-loss	40.57%	70.25%	94.73%	68.23%

**Table 8:** The ablation study of AEN [58] from BMN [41] with C3D using $AR@A_{N}$ in THUMOS 2014.

Methods	AR@50	AR@100	AR@200	AR@500	AR@1000
BMN	32.73%	40.68%	47.86%	56.42%	60.44%
BMN with AEN	33.26%	42.93%	50.32%	59.10%	64.03%

## Data Availability

The ActivityNet v1.3 dataset is openly available at http://www.activity-net.org/ (accessed on 2 August 2021), reference number [11] and THUMOS 2014 dataset is openly available at http://crcv.ucf.edu/THUMOS14/download.html (accessed on 21 April 2022), reference number [9].

## References

[1] Ji J., Cao K., Niebles J.C. Learning temporal action proposals with fewer labels. Proceedings of the IEEE/CVF International Conference on Computer Vision.

[2] Wang X., Zhang S., Qing Z., Shao Y., Gao C., Sang N. Self-supervised learning for semi-supervised temporal action proposal. Proceedings of the IEEE/CVF Conference on Computer Vision and Pattern Recognition.

[3] Tan J., Tang J., Wang L., Wu G. Relaxed transformer decoders for direct action proposal generation. Proceedings of the IEEE/CVF International Conference on Computer Vision.

[4] Chang S., Wang P., Wang F., Li H., Feng J. (2021). Augmented Transformer with Adaptive Graph for Temporal Action Proposal Generation. arXiv.

[5] Girshick R. Fast r-cnn. Proceedings of the IEEE International Conference on Computer Vision.

[6] Schindler K., Van Gool L. Action snippets: How many frames does human action recognition require?. Proceedings of the 2008 IEEE Conference on Computer Vision and Pattern Recognition.

[7] Satkin S., Hebert M. (2010). Modeling the temporal extent of actions. European Conference on Computer Vision.

[8] Xia H., Zhan Y. (2020). A Survey on Temporal Action Localization. IEEE Access.

[9] Idrees H., Zamir A.R., Jiang Y.G., Gorban A., Laptev I., Sukthankar R., Shah M. (2017). The THUMOS challenge on action recognition for videos “in the wild”. Comput. Vis. Image Underst..

[10] Soomro K., Zamir A.R., Shah M. (2012). UCF101: A dataset of 101 human actions classes from videos in the wild. arXiv.

[11] Caba Heilbron F., Escorcia V., Ghanem B., Carlos Niebles J. Activitynet: A large-scale video benchmark for human activity understanding. Proceedings of the IEEE Conference on Computer Vision and Pattern Recognition.

[12] Stoian A., Ferecatu M., Benois-Pineau J., Crucianu M. (2015). Fast action localization in large-scale video archives. IEEE Trans. Circuits Syst. Video Technol..

[13] Yeung S., Russakovsky O., Jin N., Andriluka M., Mori G., Fei-Fei L. (2018). Every moment counts: Dense detailed labeling of actions in complex videos. Int. J. Comput. Vis..

[14] Sigurdsson G.A., Varol G., Wang X., Farhadi A., Laptev I., Gupta A. (2016). Hollywood in homes: Crowdsourcing data collection for activity understanding. European Conference on Computer Vision.

[15] Gu C., Sun C., Ross D.A., Vondrick C., Pantofaru C., Li Y., Vijayanarasimhan S., Toderici G., Ricco S., Sukthankar R. Ava: A video dataset of spatio-temporally localized atomic visual actions. Proceedings of the IEEE Conference on Computer Vision and Pattern Recognition.

[16] Karpathy A., Toderici G., Shetty S., Leung T., Sukthankar R., Fei-Fei L. Large-scale video classification with convolutional neural networks. Proceedings of the IEEE Conference on Computer Vision and Pattern Recognition.

[17] Donahue J., Anne Hendricks L., Guadarrama S., Rohrbach M., Venugopalan S., Saenko K., Darrell T. Long-term recurrent convolutional networks for visual recognition and description. Proceedings of the IEEE Conference on Computer Vision and Pattern Recognition.

[18] Yue-Hei Ng J., Hausknecht M., Vijayanarasimhan S., Vinyals O., Monga R., Toderici G. Beyond short snippets: Deep networks for video classification. Proceedings of the IEEE Conference on Computer Vision and Pattern Recognition.

[19] Ji S., Xu W., Yang M., Yu K. (2012). 3D convolutional neural networks for human action recognition. IEEE Trans. Pattern Anal. Mach. Intell..

[20] Taylor G.W., Fergus R., LeCun Y., Bregler C. (2010). Convolutional learning of spatio-temporal features. European Conference on Computer Vision.

[21] Tran D., Bourdev L., Fergus R., Torresani L., Paluri M. Learning spatiotemporal features with 3d convolutional networks. Proceedings of the IEEE International Conference on Computer Vision.

[22] Varol G., Laptev I., Schmid C. (2017). Long-term temporal convolutions for action recognition. IEEE Trans. Pattern Anal. Mach. Intell..

[23] Simonyan K., Zisserman A. Two-stream convolutional networks for action recognition. Proceedings of the Neural Information Processing Systems (NIPS).

[24] Li Q., Yang W., Chen X., Yuan T., Wang Y. (2020). Temporal Segment Connection Network for Action Recognition. IEEE Access.

[25] Carreira J., Zisserman A. Quo vadis, action recognition? a new model and the kinetics dataset. Proceedings of the IEEE Conference on Computer Vision and Pattern Recognition.

[26] Huang Y., Guo Y., Gao C. (2020). Efficient parallel inflated 3D convolution architecture for action recognition. IEEE Access.

[27] Alwassel H., Giancola S., Ghanem B. Tsp: Temporally-sensitive pretraining of video encoders for localization tasks. Proceedings of the IEEE/CVF International Conference on Computer Vision.

[28] Gao J., Yang Z., Chen K., Sun C., Nevatia R. Turn tap: Temporal unit regression network for temporal action proposals. Proceedings of the IEEE International Conference on Computer Vision.

[29] Buch S., Escorcia V., Shen C., Ghanem B., Carlos Niebles J. Sst: Single-stream temporal action proposals. Proceedings of the IEEE conference on Computer Vision and Pattern Recognition.

[30] Huang J., Li N., Zhang T., Li G., Huang T., Gao W. Sap: Self-adaptive proposal model for temporal action detection based on reinforcement learning. Proceedings of the Thirty-Second AAAI Conference on Artificial Intelligence.

[31] Eun H., Lee S., Moon J., Park J., Jung C., Kim C. (2019). Srg: Snippet relatedness-based temporal action proposal generator. IEEE Trans. Circuits Syst. Video Technol..

[32] Hu Y., Jin Y., Li R., Zhang X. (2019). CMSN: Continuous Multi-stage Network and Variable Margin Cosine Loss for Temporal Action Proposal Generation. arXiv.

[33] Gao J., Shi Z., Wang G., Li J., Yuan Y., Ge S., Zhou X. Accurate temporal action proposal generation with relation-aware pyramid network. Proceedings of the AAAI Conference on Artificial Intelligence.

[34] He K., Zhang X., Ren S., Sun J. Deep residual learning for image recognition. Proceedings of the IEEE Conference on Computer Vision and Pattern Recognition.

[35] Mnih V., Kavukcuoglu K., Silver D., Rusu A.A., Veness J., Bellemare M.G., Graves A., Riedmiller M., Fidjeland A.K., Ostrovski G. (2015). Human-level control through deep reinforcement learning. Nature.

[36] Zhao Y., Xiong Y., Wang L., Wu Z., Tang X., Lin D. Temporal action detection with structured segment networks. Proceedings of the IEEE International Conference on Computer Vision.

[37] Carion N., Massa F., Synnaeve G., Usunier N., Kirillov A., Zagoruyko S. (2020). End-to-end object detection with transformers. European Conference on Computer Vision.

[38] Li L., Kong T., Sun F., Liu H. (2019). Deep point-wise prediction for action temporal proposal. International Conference on Neural Information Processing.

[39] Lin C., Li J., Wang Y., Tai Y., Luo D., Cui Z., Wang C., Li J., Huang F., Ji R. Fast learning of temporal action proposal via dense boundary generator. Proceedings of the AAAI Conference on Artificial Intelligence.

[40] Lin T., Zhao X., Su H., Wang C., Yang M. Bsn: Boundary sensitive network for temporal action proposal generation. Proceedings of the European Conference on Computer Vision (ECCV).

[41] Lin T., Liu X., Li X., Ding E., Wen S. Bmn: Boundary-matching network for temporal action proposal generation. Proceedings of the IEEE/CVF International Conference on Computer Vision.

[42] Bai Y., Wang Y., Tong Y., Yang Y., Liu Q., Liu J. (2020). Boundary content graph neural network for temporal action proposal generation. European Conference on Computer Vision.

[43] Zhao P., Xie L., Ju C., Zhang Y., Wang Y., Tian Q. (2020). Bottom-up temporal action localization with mutual regularization. European Conference on Computer Vision.

[44] Roerdink J.B., Meijster A. (2000). The watershed transform: Definitions, algorithms and parallelization strategies. Fundam. Inform..

[45] Wang L., Xiong Y., Wang Z., Qiao Y., Lin D., Tang X., Van Gool L. (2018). Temporal segment networks for action recognition in videos. IEEE Trans. Pattern Anal. Mach. Intell..

[46] Xiong Y., Zhao Y., Wang L., Lin D., Tang X. (2017). A pursuit of temporal accuracy in general activity detection. arXiv.

[47] Lin T.Y., Dollár P., Girshick R., He K., Hariharan B., Belongie S. Feature pyramid networks for object detection. Proceedings of the IEEE Conference on Computer Vision and Pattern Recognition.

[48] Girdhar R., Carreira J., Doersch C., Zisserman A. Video action transformer network. Proceedings of the IEEE/CVF Conference on Computer Vision and Pattern Recognition.

[49] Gao J., Chen K., Nevatia R. Ctap: Complementary temporal action proposal generation. Proceedings of the European Conference on Computer Vision (ECCV).

[50] Liu Y., Ma L., Zhang Y., Liu W., Chang S.F. Multi-granularity generator for temporal action proposal. Proceedings of the IEEE/CVF Conference on Computer Vision and Pattern Recognition.

[51] Wang L., Yang H., Wu W., Yao H., Huang H. (2021). Temporal Action Proposal Generation with Transformers. arXiv.

[52] Vaswani A., Shazeer N., Parmar N., Uszkoreit J., Jones L., Gomez A.N., Kaiser Ł., Polosukhin I. (2017). Attention is all you need. Adv. Neural Inf. Process. Syst..

[53] Soomro K., Shah M. Unsupervised action discovery and localization in videos. Proceedings of the IEEE International Conference on Computer Vision.

[54] Tarvainen A., Valpola H. (2017). Mean teachers are better role models: Weight-averaged consistency targets improve semi-supervised deep learning results. arXiv.

[55] Lin J., Gan C., Han S. Tsm: Temporal shift module for efficient video understanding. Proceedings of the IEEE/CVF International Conference on Computer Vision.

[56] Liu S., Zhao X., Su H., Hu Z. TSI: Temporal Scale Invariant Network for Action Proposal Generation. Proceedings of the Asian Conference on Computer Vision.

[57] Su H., Gan W., Wu W., Qiao Y., Yan J. Bsn++: Complementary boundary regressor with scale-balanced relation modeling for temporal action proposal generation. Proceedings of the AAAI Conference on Artificial Intelligence.

[58] Vo-Ho V.K., Le N., Kamazaki K., Sugimoto A., Tran M.T. Agent-Environment Network for Temporal Action Proposal Generation. Proceedings of the ICASSP 2021—2021 IEEE International Conference on Acoustics, Speech and Signal Processing (ICASSP).

[59] Feichtenhofer C., Fan H., Malik J., He K. Slowfast networks for video recognition. Proceedings of the IEEE/CVF International Conference on Computer Vision.

[60] Rendón-Segador F.J., Álvarez-García J.A., Enríquez F., Deniz O. (2021). ViolenceNet: Dense Multi-Head Self-Attention with Bidirectional Convolutional LSTM for Detecting Violence. Electronics.

[61] Song L., Zhang S., Yu G., Sun H. Tacnet: Transition-aware context network for spatio-temporal action detection. Proceedings of the IEEE/CVF Conference on Computer Vision and Pattern Recognition.

[62] Wray M., Doughty H., Damen D. On Semantic Similarity in Video Retrieval. Proceedings of the IEEE/CVF Conference on Computer Vision and Pattern Recognition.

[63] Patrick M., Huang P.Y., Asano Y., Metze F., Hauptmann A., Henriques J., Vedaldi A. (2020). Support-set bottlenecks for video-text representation learning. arXiv.

[64] Wray M., Larlus D., Csurka G., Damen D. Fine-grained action retrieval through multiple parts-of-speech embeddings. Proceedings of the IEEE/CVF International Conference on Computer Vision.

[65] Xiong B., Kalantidis Y., Ghadiyaram D., Grauman K. Less is more: Learning highlight detection from video duration. Proceedings of the IEEE/CVF Conference on Computer Vision and Pattern Recognition.

[66] Xu M., Wang H., Ni B., Zhu R., Sun Z., Wang C. Cross-category Video Highlight Detection via Set-based Learning. Proceedings of the IEEE/CVF International Conference on Computer Vision.

[67] Ye Q., Shen X., Gao Y., Wang Z., Bi Q., Li P., Yang G. Temporal Cue Guided Video Highlight Detection With Low-Rank Audio-Visual Fusion. Proceedings of the IEEE/CVF International Conference on Computer Vision.

[68] Apostolidis E., Adamantidou E., Metsai A.I., Mezaris V., Patras I. (2021). Video Summarization Using Deep Neural Networks: A Survey. arXiv.

[69] Saquil Y., Chen D., He Y., Li C., Yang Y.L. Multiple Pairwise Ranking Networks for Personalized Video Summarization. Proceedings of the IEEE/CVF International Conference on Computer Vision.

[70] Dosovitskiy A., Beyer L., Kolesnikov A., Weissenborn D., Zhai X., Unterthiner T., Dehghani M., Minderer M., Heigold G., Gelly S. (2020). An image is worth 16x16 words: Transformers for image recognition at scale. arXiv.

[71] Aytar Y., Vondrick C., Torralba A. (2016). Soundnet: Learning sound representations from unlabeled video. Adv. Neural Inf. Process. Syst..

